# The Eurofever Project: towards the longitudinal stage

**DOI:** 10.1186/1546-0096-13-S1-P182

**Published:** 2015-09-28

**Authors:** S Federici, J Frenkel, S Ozen, M Finetti, F Garibotto, H Lachmann, A Martini, N Ruperto

**Affiliations:** 1 Gaslini Institute, II Division of Pediatric, Genova, Italy; 2 University Medical Center Utrecht, Department of Paediatrics, Utrecht, the Netherlands; 3 Hacettepe University, Department of Pediatric Nephrology and Rheumatology, Ankara, Turkey; 4 University College Medical School, National Amyloidosis Centre, Royal Free Campus, London, UK

## Background

In 2008 the Paediatric Rheumathology European Society (PReS) promoted an International Project for the study of Autoinflammatory Diseases (AID) named Eurofever whose main purpose is to create a web-based registry for the collection of clinical, laboratory and response to treatment information in patients with AID.

## Objectives

To implement the Registry with the new recently described AID and genes and modify the web-system to make it suitable for the collection of longitudinal data.

## Methods

With the technical help of Paediatric Rheumatology INternational Trial Organization (PRINTO) web-masters, we were able to revise the electronic case report forms bringing 2 main novelties: i) inclusion of the more recently described AID (DADA2, CAMPS, SAVI) and the relative clinical manifestation ii) modification of drug layout. In this process information on safety and efficacy has been included. Very soon Centres will be asked to update on a yearly base the information regarding each patient with particular focus on modification of the clinical picture, onset of complication/sequelae, treatment, adverse events if present, laboratory and instrumental findings.

## Results

At present baseline demographic information from 3089 (M:F=1513:1576) patients from 101 centers in 38 countries are available. In 77% complete clinical information from disease onset to diagnosis and response to treatment is also available. For each disease the number of enrolled patients is: FMF 894 pts (708 with complete clinical data); TRAPS 268 pts (226 complete); CAPS 284 pts (208 complete); MKD 189 pts (165 complete); Blau's disease 71 pts (22 complete); PAPA 27 pts (22 complete); NLRP-12 mediated periodic fever 14 pts (9 complete); DIRA 3 pts (all complete); CANDLE 2 pts (1 complete), Schnitzler 1 pt and Majeed 2 pts (both complete). Among multifactorial autoinflammatory diseases: PFAPA 612 pts (380 complete); CRMO 417 pts (394 complete); pediatric Bechet disease 92 pts (72 complete) and 217 patients with undefined periodic fever (182 complete). Longitudinal electronic data capture is now ready and online for authorized PRINTO members.

## Conclusions

A common registry for collection of patients with AID is available and the enrollment is ongoing. This project represents the first attempt of a common registry for different autoinflammatory syndromes. The longitudinal collection and analysis of data coming from the Registry will improve our knowledge in the field of autoinflammation both on the natural history of the single disease and the efficacy and safety of the treatment commonly used in the clinical practice with particular regards to biologics.

